# CDK4/6 Inhibitor-Induced Senescence in Cancer: Mechanisms and Therapeutic Implications

**DOI:** 10.3390/cancers18142192

**Published:** 2026-07-08

**Authors:** Simin Elif Türker, Marco Demaria, Boshi Wang

**Affiliations:** European Research Institute for the Biology of Ageing (ERIBA), University Medical Center Groningen (UMCG), University of Groningen (RUG), 9713 AV Groningen, The Netherlands

**Keywords:** CDK4/6 inhibitors, senescence, SASP, combination therapies

## Abstract

Pharmacological cyclin-dependent kinase 4 and 6 (CDK4/6) inhibitors have reshaped the treatment landscape of HR-positive, HER2-negative (HR+/HER2−) breast cancer and are increasingly being explored across diverse malignancies. By preventing retinoblastoma (RB) phosphorylation and enforcing G1-S cell cycle arrest, these agents achieve durable tumour control with a more favourable toxicity profile than conventional chemotherapy. Beyond their canonical cytostatic effects, prolonged CDK4/6 inhibitor treatments induce cellular senescence, a stable, proliferative arrest accompanied by profound transcriptional, epigenetic, and secretory changes. This review summarises current knowledge on CDK4/6 inhibitor-induced senescence in both cancer and normal cells as a central biological mechanism that links tumour suppression and microenvironmental remodelling. Importantly, this process is highly context-dependent, differing between tumour and non-malignant cells, with a distinct senescence-associated secretory phenotype (SASP) that shapes immune responses and tissue homeostasis. We also discuss how CDK4/6 inhibitor-induced senescence influences the tumour microenvironment by modulating immune surveillance, stromal interactions, and cancer cell plasticity. Finally, we examine emerging resistance mechanisms and rational combination strategies for CDK4/6 inhibitors, including targeting compensatory signalling pathways, immune checkpoint blockades, and senescence-directed sequential therapies. Collectively, CDK4/6 inhibitor-induced senescence represents both a challenge and a therapeutic opportunity, underscoring the need to integrate cell cycle control with the modulation of cellular states.

## Simple Summary

CDK4/6 inhibitors are a class of targeted anticancer drugs that block cell division and are widely used to treat a broad range of malignancies. Beyond these antiproliferative effects, prolonged CDK4/6 inhibition can cause both cancer and normal cells to enter a state called cellular senescence in which cells stably stop dividing but remain metabolically active. In this review, we examine how CDK4/6 inhibitor-induced senescence reshapes the tumour microenvironment and modulates antitumour immune responses. We also discuss how these senescence-associated changes can be therapeutically exploited to improve cancer treatment.

## 1. Introduction

Cyclin-dependent kinase 4 and 6 (CDK4/6) inhibitors are a class of targeted cancer therapeutic drugs that suppress cell cycle progression by preventing retinoblastoma (RB) protein phosphorylation and G1-S phase transition [1]. Agents in this class include U.S. FDA-approved palbociclib, ribociclib, and abemaciclib, as well as trilaciclib, which is approved for myeloprotection, and emerging CDK4/6 inhibitors, such as dalpiciclib and atirmociclib [2,3,4,5]. These agents are commonly used as standalone therapies or in combination with endocrine therapies in hormone receptor-positive (HR^+^) and HER2-negative (HER2^−^) breast cancers [6,7,8]. In addition, they are actively being investigated in other malignancies such as melanoma, non-small cell lung cancer, and other solid tumours, reflecting their broader therapeutic potential beyond breast cancer treatment [9,10,11,12,13]. CDK4/6 inhibitors exhibit a more favourable toxicity profile than conventional genotoxic chemotherapy, which is often associated with substantial treatment burden, while CDK4/6 inhibitors have demonstrated improved clinical outcomes in selected patient populations [14,15,16]. Notably, their tolerability allows prolonged administration, as illustrated by clinical studies in which abemaciclib has been administered continuously for up to two years in the adjuvant setting to reduce the risk of relapse [17].

Beyond their intended therapeutic effects, CDK4/6 inhibitors (CDK4/6i) have been shown to induce cellular senescence upon long-term treatment. Senescence is defined as a state of stable proliferative arrest in which cells permanently exit the cell cycle without undergoing cell death [18,19,20]. While traditionally considered irreversible, emerging evidence suggests that under certain conditions, therapy-induced senescent cancer cells may escape this arrest and re-enter the cell cycle, as discussed further in subsequent sections [21,22]. Senescent cells are characterised by elevated senescence-associated β-galactosidase (SA-β-gal) activity, elevated expression of cell cycle inhibitors such as p16^INK4a^, p19^INK4d^, p21^CIP1^, and p53, and the development of a senescence-associated secretory phenotype (SASP) [23,24]. It should be noted that senescence markers vary across studies, and no single marker is sufficient for definitive identification. A combination of markers, including SA-β-gal activity, p16^INK4a^, p21^CIP1^ expression, and SASP profiling, is recommended in accordance with current guidelines [19]. The SASP is highly heterogeneous and varies depending on the inducing stimulus, cell type, and tissue context. As a major mediator of the paracrine effects of senescent cells, the SASP plays a central role in shaping the tumour microenvironment [25]. Importantly, CDK4/6 inhibitors induce senescence in normal stromal cells with a functionally distinct SASP profile [26,27]. Interestingly, CDK4/6 inhibitors can also suppress pre-existing SASP factors in senescent cells, highlighting the multifaceted and context-dependent role of CDK4/6 signalling in regulating therapy-induced senescence programs [28].

Therapy-induced senescence does not eliminate tumour cells, and residual cell populations may persist within the tumour microenvironment and contribute to cancer relapse [29]. A comparable phenomenon is observed following chemotherapy, where treated tumour cell populations become enriched in stem-cell-like subpopulations, commonly referred to as cancer stem cells (CSCs), which can also drive cancer relapse following treatment [30,31]. Interestingly, emerging evidence suggests that senescent cancer cells may acquire stem-cell-like properties [32,33]. This phenotype is associated with enhanced tumour cellular plasticity, metastatic potential, and resistance to therapy [34]. The effects of CDK4/6 inhibitors on cancer stem cell behaviour remain incompletely understood, and the current evidence has yet to be comprehensively synthesised.

This narrative review specifically focuses on CDK4/6 inhibitor-induced senescence as a distinct biological state, positioning it as a unifying axis connecting tumour cell cycle control, microenvironmental remodelling, and rational combination strategies and examining its context-dependent consequences across tumour and normal cells.

## 2. Clinical Landscape of CDK4/6 Inhibitors in Cancer Therapy

The clinical application of CDK4/6 inhibitors has expanded since their initial approval, becoming a standard component of therapy for HR^+^/HER2^−^ breast cancer. The use of CDK4/6 inhibitors in combination with endocrine therapy became established through multiple large randomised clinical trials, demonstrating significant improvements in progression-free survival and overall survival compared to endocrine therapy alone [35,36,37,38]. These combinations typically include endocrine agents such as aromatase inhibitors or the selective oestrogen receptor degrader fulvestrant to suppress oestrogen receptor signalling [37]. As a result, CDK4/6 inhibitors have become a standard-of-care strategy in both metastatic and high-risk early-stage disease. Endocrine therapy remains the therapeutic foundation in HR^+^/HER2^−^ breast cancer, and the addition of CDK4/6 inhibitors enhances treatment efficacy by reinforcing cell cycle control and delaying the emergence of resistance [39,40]. Compared to conventional chemotherapy or endocrine therapy, CDK4/6 inhibitors are associated with a more manageable toxicity profile, with common milder side effects including neutropenia, fatigue, and gastrointestinal toxicity [41,42]. These side effects are generally reversible and can often be managed through dose adjustment or temporary treatment interruption, allowing patients to maintain therapy over extended periods [43,44]. This is particularly important in both metastatic and adjuvant settings where sustained treatment is required to control disease progression while preserving quality of life.

While the clinical benefit of CDK4/6 inhibitors is most established in HR^+^/HER2^−^ breast cancer, their therapeutic potential is being explored in other malignancies, including non-small cell lung cancer, melanoma, and selected gynaecological and gastrointestinal cancers [9,45,46,47,48,49]. However, clinical outcomes have been variable, with more success in particular combinations with targeted or immune-mediated therapies, highlighting the importance of tumour-specific context in determining response. Large randomised trials of next-generation CDK4/6 inhibitors, such as atirmociclib, are being investigated for better selectivity with less toxicity [50]. Dalpiciclib, another emerging next-generation CDK4/6 inhibitor, has also yielded favourable clinical results. In the phase 3 DAWNA-1 trial, dalpiciclib in combination with fulvestrant significantly prolonged progression-free survival in patients with advanced HR^+^/HER2^−^ breast cancer who had previously received endocrine therapy [4]. Overall, CDK4/6 inhibitors remain a cornerstone of cancer therapy, and further investigation into their mechanisms of action and rational combination strategies is warranted.

## 3. CDK4/6 Inhibitor-Induced Senescence: Mechanisms and Consequences

As prolonged CDK4/6 inhibition induces cellular senescence in both tumour and non-malignant cells, understanding the molecular mechanisms underlying this process is essential for interpreting both its therapeutic benefits and potential adverse effects [21,26,51,52,53,54].

### 3.1. Mechanisms of CDK4/6-Induced Senescence

The primary trigger for CDK4/6 inhibition-induced cell cycle arrest is RB hypo-phosphorylation, which leads to the sustained repression of E2F transcription factors and downregulation of genes involved in DNA replication and mitotic progression [55]. With prolonged treatment, this durable arrest can progress to senescence, accompanied by the activation of tumour-suppressive pathways, particularly p53/p21^CIP^ and p16^INK4a^, as well as broader transcriptional and chromatin remodelling that persists even after drug withdrawal [20,26,51,56]. These changes indicate that CDK4/6 inhibitor-induced senescence represents an actively stabilised cellular state rather than a passive form of cell cycle arrest dependent on continuous drug exposure.

**Figure 1:**
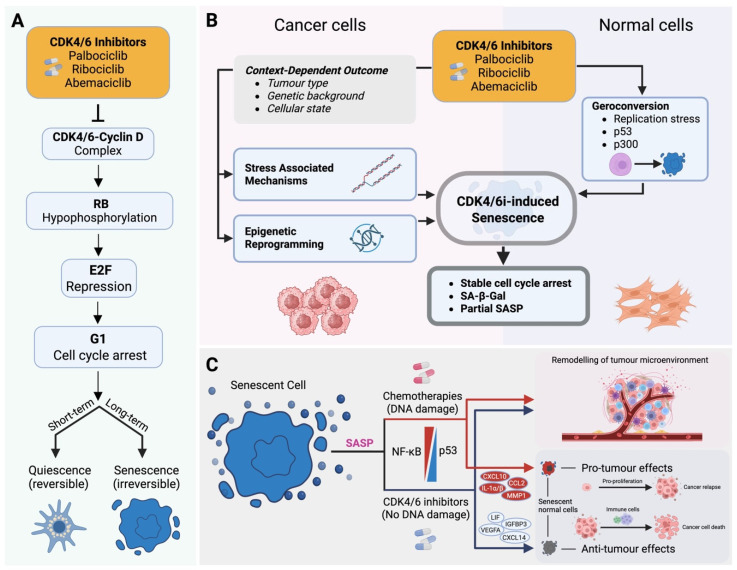
CDK4/6 inhibitor-induced senescence: mechanisms and consequences. (**A**) CDK4/6 inhibitors (palbociclib, ribociclib, and abemaciclib) inhibit the CDK4/6-Cyclin D complex, leading to RB hypophosphorylation, the repression of E2F activity, and the induction of G1 cell cycle arrest. Depending on treatment duration and cellular context, cells undergo a fate decision between reversible quiescence and stable irreversible senescence. (**B**) CDK4/6 inhibitor-induced senescence is established through multiple interconnected mechanisms. In cancer cells, these include epigenetic reprogramming (e.g., DNMT1 downregulation, reduced DNA methylation, and chromatin remodelling) and stress-associated processes (replication stress, impaired origin licensing, metabolic stress, and cellular overgrowth). These mechanisms are context-dependent and influenced by tumour type, genetic background, and cellular state. In normal cells, p53-associated geroconversion contributes to the transition from reversible arrest to senescence. Upon senescence induction, tumour suppressor pathways (p53/p21 and p16^Ink4a^/RB) are activated, SA-β-Gal activity is increased, and a partial SASP is established. CDK4/6i, CDK4/6 inhibitors: (**C**) cancer therapy-induced senescent cells secrete a senescence-associated secretory phenotype (SASP), mediating downstream effects on tumour progression. In cancer cells, SASP factors remodel the tumour microenvironment and influence disease outcomes. In normal cells, SASP composition varies depending on the inducing stimulus. Chemotherapy-induced senescence (DNA damage-driven) is associated with a robust NF-κB-dependent pro-inflammatory SASP, whereas CDK4/6 inhibitor-induced senescence (DNA damage-independent) exhibits a more restricted, context-dependent profile linked to p53 signalling. Chemotherapy-induced senescence is characterised by the increased secretion of inflammatory cytokines, chemokines, and matrix-remodelling factors (including IL-1α/β, CCL2, CXCL10, and MMP1), contributing to pro-tumour effects such as chronic inflammation and cancer relapse. In contrast, CDK4/6 inhibitor-induced senescence is associated with p53/p21-regulated factors (such as CXCL14, IGFBP3, VEGFA, and LIF), which promote anti-tumour effects including immune-mediated clearance. Both act in a paracrine manner.

In tumour cells, CDK4/6 inhibitor-induced senescence is not a uniform consequence of G1 phase cell cycle arrest but, rather, a context-dependent outcome shaped by tumour-specific molecular features. While RB/E2F repression provides the initiating signal, additional mechanisms determine whether cells remain in reversible arrest (quiescence) or transition to irreversible senescence, a process influenced by the duration of drug treatment (Figure 1A). Upon prolonged drug exposure, epigenetic regulation emerges as a key determinant: CDK4/6 inhibition has been linked to the autophagy-dependent degradation of DNMT1, leading to the loss of the DNA methylation maintenance and transcriptional reprogramming that promotes senescence [57]. In parallel, the downregulation of mitotic transcription factors such as FOXM1 further reinforces exit from proliferative programs [58]. Chromatin regulators, including ATRX and RB-dependent remodelling processes, have also been implicated in establishing a permissive chromatin state that stabilises the senescence-associated transcriptional profiles (Figure 1B) [59]. Another key mechanism involves p53-dependent signalling, since recent studies have suggested that prolonged CDK4/6 inhibition may drive a TP53-mediated “geroconversion” in which sustained arrest is actively converted to a stable senescent state rather than remaining reversible (Figure 1B) [60,61]. Additional tumour-specific mechanisms, including p300-mediated p53 acetylation and regulation of MDM2 turnover, further support the role of p53 activation in reinforcing CDK4/6 inhibition-induced senescence (Figure 1B) [62,63]. In addition, replication-associated and metabolism stress appear to contribute to this transition [52]. Prolonged CDK4/6 inhibition has been associated with impaired origin licensing, replication stress, and cellular overgrowth, suggesting that senescence may arise when tumour cells fail to maintain homeostasis under sustained cell cycle blockades (Figure 1B) [52,64]. These mechanisms provide a link between cytostatic arrest and irreversible state transition, which mark the establishment of senescence. Furthermore, these pathways are not mutually exclusive and may act in combination. The relative contribution of each mechanism is likely to depend on tumour type, genetic background, and cellular state, which may explain the variability in senescence induction mechanisms observed across different cancer models, including breast cancer, melanoma, sarcoma, neuroblastoma, and ovarian cancer (Figure 1B) [58,62,65,66,67]. Overall, these findings indicate that CDK4/6 inhibitor-induced senescence in tumour cells represents a multi-layered process integrating cell cycle inhibition with epigenetic regulation, replication stress mechanisms, and tumour-specific signalling networks.

In non-malignant normal cells, prolonged treatment with CDK4/6 inhibitors can also induce senescence, but this appears to be mechanistically and functionally different from tumour cell senescence. Pharmacological CDK4/6 inhibition has been shown to induce a p53-dependent senescent state in non-malignant normal cells (Figure 1B), supporting the idea that senescence can directly arise from prolonged cell cycle arrest even in the absence of oncogenic and genotoxic stresses [26]. Unlike classical DNA-damage-induced senescence, this state is characterised by a comparatively restrained inflammatory profile, with a restricted SASP dependent on p53 signalling rather than NF-κB-driven pro-inflammatory components (Figure 1B,C) [25,26]. This restricted SASP suggests that CDK4/6 inhibitor-induced senescence in normal cells represents a distinct DNA damage-free biological state from senescent cells induced by other conventional therapies. Prolonged CDK4/6 inhibition in non-malignant cells has also been shown to be related to osmotic imbalance, cellular overgrowth, and replication-associated stress, providing insights into how sustained arrest can transition to senescence in the absence of oncogenic or genotoxic stresses (Figure 1) [52,64]. Notably, senescence in non-malignant cells has implications beyond cell-intrinsic effects. Senescent stromal and endothelial cells can influence the tumour microenvironment through altered cytokine signalling, immune modulation, and tissue remodelling [68]. Emerging evidence suggests senescence in these cells may contribute to changes in metastatic dynamics and immune responses, indicating that normal cell senescence may indirectly affect tumour progression and treatment outcomes.

### 3.2. CDK4/6 Inhibitor-Induced SASP and Microenvironmental Remodelling

The downstream effects of cancer therapy-induced senescent cancer and normal cells are largely mediated through the senescence-associated secretory phenotype (SASP). The SASP comprises a broad range of secreted factors, including cytokines, chemokines, proteases, and extracellular matrix components whose composition is highly context-dependent, varying with the inducing stimulus, cell type, and tissue environment (Figure 1C) [69,70]. The SASP initially exerts anti-cancer effects by reinforcing senescence, activating the immune system and promoting tissue repair [71]. When senescence persists, the sustained secretion of these factors can modulate cellular communication, promote chronic inflammation and facilitate adaptation to immune pressure, contributing to tumour progression [69,71].

The composition of the SASP varies between malignant and non-malignant normal cells following prolonged CDK4/6 inhibition. It also differs according to the specific pharmacological inhibitors used, with distinct profiles observed in senescent cancer cells induced by agents such as palbociclib and abemaciclib. For example, SASP components including CCL2 and CXCL10 were upregulated in palbociclib-induced senescent breast cancer cells [72]. These chemokines can recruit immune cells, including macrophages, natural killer cells, T lymphocytes, and neutrophils, to the tumour microenvironment, contributing to the immune-mediated clearance of senescent cells [72,73]. In contrast, abemaciclib has been reported to induce senescence in breast cancer cells without the robust induction of those pro-inflammatory SASP factors, yet it still elicits anti-tumour immune responses by suppressing the proliferation of regulatory T cells, showing the engagement of alternative immune-modulatory mechanisms [74]. While this immune recruitment may support the clearance of senescent tumour cells, sustained SASP signalling in tumour cells may also promote chronic inflammation, immune modulation, and tumour progression [25].

In contrast, the SASP profile in CDK4/6 inhibitor-induced senescent normal cells has been described as partial [26]. This profile is enriched in p53-associated signalling, but it lacks the detrimental NF-κB-driven pro-inflammatory components that are typically observed in classical senescence (Figure 1C) [26,75]. As a result, this restricted SASP may retain the ability to promote paracrine senescence and undergo more efficient clearance without the pro-tumourigenic and detrimental effects of inflammatory signalling [26]. This suggests that CDK4/6 inhibitor-induced senescence in normal cells may support tissue homeostasis and controlled immune engagement rather than persistent inflammatory signalling linked to tumour progression. These distinct SASP profiles carry different immune implications: chemotherapy-induced senescence drives robust NF-κB-dependent inflammation with pro-tumourigenic potential, CDK4/6 inhibitor-induced senescence in tumour cells elicits selective immune recruitment, and CDK4/6 inhibitor-induced senescence in normal cells engages a restricted p53-dependent secretome associated with controlled immune clearance.

## 4. CDK4/6 Inhibitors and the Tumour Microenvironment

CDK4/6 inhibitors influence multiple cellular and non-cellular components within the tumour microenvironment through both direct and senescence-mediated mechanisms [76]. The tumour microenvironment comprises immune cells, endothelial cells, stromal fibroblasts, extracellular matrix components, and heterogeneous tumour cell subpopulations, all of which dynamically interact to regulate tumour progression and therapeutic response [77,78]. Increasing evidence suggests that CDK4/6 inhibitors reshape this complex environment through the modulation of immune signalling, cellular plasticity, and senescence-associated secretory phenotypes [51,79,80].

### 4.1. Effects on Healthy Normal Cells

**Figure 2:**
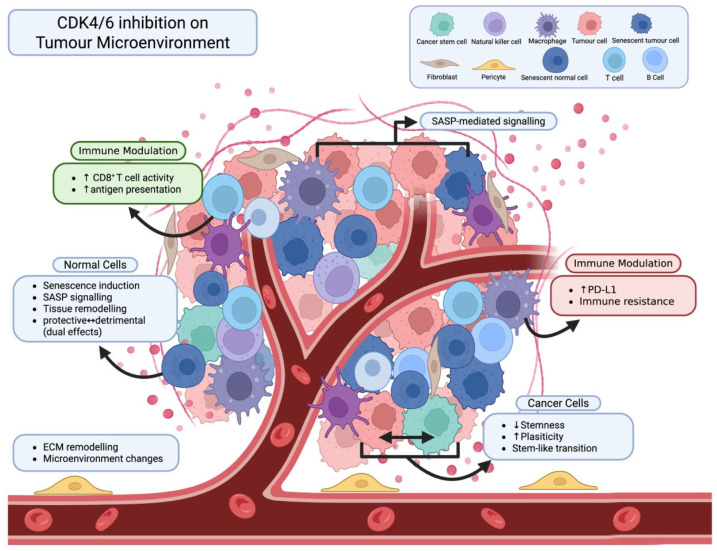
CDK4/6 inhibitors and the tumour microenvironment. CDK4/6 inhibition in tumour cells induces senescence and promotes the release of senescence-associated secretory phenotype (SASP) factors, which modulate multiple components of the tumour microenvironment. CDK4/6 inhibitors also influence cancer cell plasticity, reducing stemness in some contexts while promoting phenotypic reprogramming and stem-like transitions under therapeutic pressure. In immune cells, CDK4/6 inhibition enhances anti-tumour immunity by increasing CD8^+^ T cell activity and antigen presentation but also by promoting adaptive immune resistance via the upregulation of PD-L1 and immune suppressive signalling. In normal cells, including fibroblasts, pericytes, and other stromal populations, CDK4/6 inhibition can induce senescence and a partial SASP profile, leading to tissue remodelling and predominantly protective effects. Additional extracellular matrix remodelling (ECM) contributes to changes in tissue architecture and intercellular communication.

One of the components within the tumour microenvironment is healthy normal cells, including endothelial cells, epithelial cells, and stromal fibroblasts. Recent research suggests that the inhibition of CDK4/6 has been reported to induce premature senescence in human fibroblasts and in mouse models [26]. Furthermore, CDK4/6 inhibition can induce cellular overgrowth associated with osmotic and replicative stress, promoting senescence in retinal epithelial pigment cells [52]. Several studies report CDK4/6 inhibition-related potential cardiovascular, haematological, and renal toxicities [81,82]. However, CDK4/6 inhibitors have also been reported to have a tumour-suppressive and protective potential on normal tissue through the activation of the RB/E2F signalling pathway while maintaining genomic stability [83]. Although these effects are described as side effects of treatment, they support the concept that CDK4/6 inhibitors exert biologically meaningful effects on healthy normal cells (Figure 2).

CDK4/6 inhibitor-induced senescence in normal cells may also modulate the tumour microenvironment through SASP-mediated signalling [26,84]. In this context, senescent stromal fibroblasts and endothelial cells can secrete cytokines, chemokines, and extracellular matrix remodelling factors that alter tissue architecture and intercellular communication. Notably, the SASP profile induced by CDK4/6 inhibition differs from that triggered by conventional chemotherapy, potentially leading to distinct effects on immune cell recruitment, vascular function, and stromal organisation (Figure 2). These changes do not necessarily promote tumour progression and might support the tumour-suppressive microenvironments in some contexts [26]. Whether senescent stromal cells support or undermine tumour control depends primarily on the nature of the inducing stimulus and the resulting SASP composition. As a result, the impact of CDK4/6 inhibition on normal tissues extends beyond toxicity, contributing to the dynamic regulation of tumour–host interactions.

### 4.2. Effects on Immune Cells

Among these components, immune cells represent a critical mediator of therapeutic response and tumour control. CDK4/6 inhibitors have been shown to enhance tumour immunogenicity through the upregulation of antigen presentation machinery and increased expression of MHC-I molecules on tumour cells, independent of senescence induction [74,85]. The suppression of E2F-driven transcription can induce interferon signalling through an innate dsRNA response linked to endogenous retroviral expression, independent of senescence, thereby promoting the expression of genes involved in antigen processing and presentation. Interferon secretion subsequently activates JAK-STAT signalling, inducing interferon-stimulated gene expression that reinforces tumour immunogenicity [86,87]. Overall, these changes increase tumour cell recognition and cytotoxic activity by CD8^+^ T lymphocytes (Figure 2) [87,88]. In addition to enhancing tumour immunogenicity, CDK4/6 inhibitors have also been reported to suppress the proliferation of regulatory T cells (Tregs), independent of senescence, a key immunosuppressive population within the tumour microenvironment [74,89]. Tregs rely on CDK4/6-mediated cell-cycle progression, and their inhibition may preferentially restrict Treg expansion relative to effector T cells [89,90]. This shift can alter the immune balance toward a more immunostimulatory state, potentially augmenting anti-tumour immune responses [90]. However, immune modulation following CDK4/6 inhibition is context dependent. While enhanced cytotoxic T cell activity may improve tumour control, the compensatory upregulation of immune checkpoint molecules such as PD-L1, which may occur both directly and via SASP-mediated signalling, and increased inflammatory signalling may also contribute to adaptive immune resistance or immune-related toxicities (Figure 2) [76,91]. These findings demonstrate the complex and dynamic effects of CDK4/6 inhibitors on anti-tumour immunity.

In parallel, CDK4/6 inhibition-induced senescence in cancer cells and, independently, in normal stromal cells within the tumour microenvironment may also modulate the immune system through distinct mechanisms (Figure 2). Senescent cells can influence immune cell recruitment and activation through the secretion of inflammatory mediators. These changes have been associated with altered leukocyte migration, immune cell composition, and enhanced tumour cell–stromal interactions [68,92]. CDK4/6 inhibitors modulate anti-tumour immunity through both direct effects on tumour cells and indirect senescence-associated mechanisms originating from both tumour and normal stromal cells within the tumour microenvironment.

### 4.3. Effects on Cancer Stem Cells

Cancer stem cells (CSCs) are tumour subpopulations with self-renewal capacity and inherent resistance to therapy, contributing to relapse and metastasis [93,94]. Emerging pre-clinical evidence suggests that CDK4/6 inhibitors may modulate CSC-associated phenotypes, although these effects appear to be highly context-dependent [95,96]. In several tumour models, CDK4/6 inhibition has been associated with the suppression of stem-like properties. For example, palbociclib has been shown to reduce sphere-forming efficiency and diminish telomerase-high, ALDH-enriched stem-like populations in lung, ovarian, and breast cancer lines [95,96]. These effects may be linked to the RB-mediated repression of E2F target genes involved in proliferation and stemness-associated transcriptional programs [97]. In addition, CDK4/6 inhibitors may interfere with the signalling pathways implicated in CSC maintenance, including WNT/β-catenin signalling, which may reduce self-renewal capacity [98].

However, the impact of CDK4/6 inhibition on CSCs is not uniformly suppressive. Increasing evidence suggests that tumour plasticity plays a role in determining CSC behaviour. Under therapeutic pressure, non-stem cancer cells may undergo phenotypic reprogramming to acquire stem-like features, potentially contributing to therapy resistance [99]. In the context of CDK4/6 inhibition, resistant tumour cells have been shown to exhibit transcriptional and signalling rewiring, reflecting adaptive changes in cellular state rather than a uniform response to therapy [22]. It is hypothesised that these adaptive responses are consistent with the increased cellular plasticity observed during CDK4/6 inhibitor treatment and may enable the emergence of stem-like phenotypes, though direct evidence in the context of CDK4/6 inhibitor-induced senescence remains limited. CDK4/6 inhibitor-induced senescence may further influence CSC behaviour. It is hypothesised that SASP factors secreted by senescent tumour and stromal cells can modulate the tumour microenvironment and influence CSC behaviour, based on evidence from broader therapy-induced senescence models [84,100]. While certain SASP components may support immune-mediated clearance and suppress tumour-initiating capacity, persistent inflammatory signalling has also been associated with the induction of stem-like phenotypes in broader senescence models, though whether CDK4/6 inhibitor-specific SASP drives similar effects remains to be established [100,101]. These findings indicate that CDK4/6 inhibitors can both suppress and indirectly promote CSC-associated traits, depending on tumour context, treatment duration, and interactions within the tumour microenvironment.

## 5. Resistance to CDK4/6 Inhibitors and Combination Therapies

**Figure 3:**
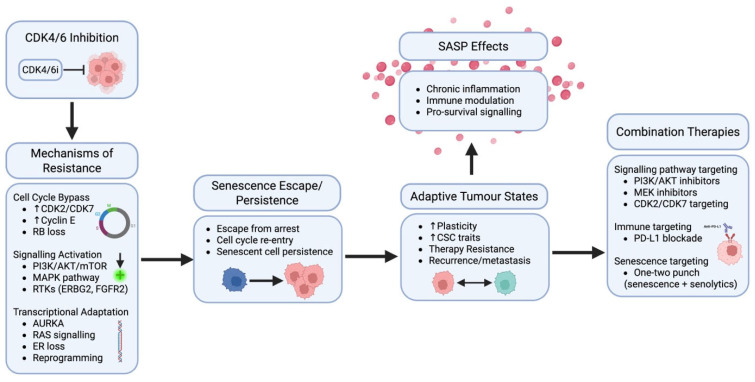
Resistance to CDK4/6 inhibitors and combination therapies. CDK4/6 inhibition initially suppresses cancer cell proliferation but promotes the emergence of resistance through multiple mechanisms. These include cell cycle bypass (CDK2/CDK7 upregulation, cyclin E increase, and RB loss), activation of compensatory signalling pathways (PI3K/AKT/mTOR, MAPK, and receptor tyrosine kinases), and transcriptional adaptation. In parallel, CDK4/6 inhibitor-induced senescence is not always stable, as tumour cells may either escape cell cycle arrest or persist in a senescent state. These processes contribute to adaptive tumour states characterised by increased cellular plasticity, cancer stem cell (CSC) traits, therapy resistance, and recurrence or metastasis. SASP factors further modulate the tumour microenvironment through chronic inflammation, immune modulation, and pro-survival signalling. These mechanisms provide a rationale for combination strategies targeting resistance pathways and cellular states. Targeting compensatory pathways, immune checkpoint inhibition, and senescence-targeting approaches such as the “one-two punch” strategy represent complementary approaches to overcome resistance and improve therapeutic outcomes.

Despite the clinical advances, resistance to CDK4/6 inhibitors inevitably emerges in a significant proportion of patients, leading to the development and clinical evaluation of combination strategies designed to target compensatory signalling pathways and overcome pathway redundancy [55]. Mechanisms of resistance include CDK2 and CDK7 upregulation, PI3K/AKT pathway activation, RB loss, loss of oestrogen receptor expression, and transcriptional reprogramming involving mitogenic and cell cycle regulators such as AKT1, RAS signalling components, aurora kinase A (AURKA), cyclin E (CCNE2), and receptor tyrosine kinases (ERBB2 and FGFR2) (Figure 3) [22,102,103,104,105,106]. These adaptive responses restore proliferative capacity and tumour plasticity.

Notably, CDK4/6-induced senescence in cancer is not always irreversible, as it has been shown that a subpopulation of cancer cells can escape the stable cell cycle arrest to re-enter the cell cycle [21,107]. This escape restores the proliferation of these cancer cells, which contributes to cancer recurrence and metastasis. Furthermore, senescent cancer cells may persist instead of being eliminated (Figure 3) [21], and over time, these persistent cells can become drug-resistant cancer cell populations. Certain SASP factors can also promote cancer cell plasticity, leading to the acquisition of stem-cell-like features as well as adaptation to therapy through the activation of pro-survival and resistance pathways [101,108]. Additionally, resistant cancer cells have been shown to exhibit increased stemness features and the activation of signalling pathways such as WNT/β-catenin, linking CSC traits to therapeutic resistance (Figure 3) [109].

Together, these resistance mechanisms illustrate the considerable plasticity of cancer cell cycle regulation and demonstrate how diverse signalling pathways can converge to restore proliferative signalling despite sustained CDK4/6 inhibition. CDK4/6 inhibition is initially cancer suppressive but can contribute to resistance in the long term through cell-intrinsic and microenvironmental mechanisms (Figure 3). These insights provide a strong mechanistic foundation for therapeutic combinations designed not only to overcome adaptive resistance but also to reshape tumour microenvironmental dynamics influenced by therapy-induced senescence.

### Mechanistic Rationale for Combination Strategies

Several of these resistance mechanisms are functionally linked to therapy-induced senescence, as SASP-mediated paracrine signalling, senescent cell persistence, and senescence escape can activate compensatory proliferative pathways including PI3K/AKT, MAPK, and alternative CDKs, providing an additional rationale for the combination strategies discussed below. The identification of compensatory signalling pathways has directly informed the design of combinatorial approaches [110]. Crosstalk between CDK4/6 signalling and the PI3K/AKT pathway represents one of the most extensively characterised resistance mechanisms. Hyperactivation of the PI3K-AKT-mTOR axis can occur through the loss of tumour suppressor PTEN or to mutations in PI3K, AKT, and mTOR, thereby promoting cell survival and proliferation despite CDK4/6 inhibition [55]. Preclinical studies have demonstrated that the dual inhibition of PI3K/AKT and CDK4/6 signalling enhances tumour suppression, reinforces cell cycle arrest, and delays resistance development [111,112,113,114]. Multiple early-phase ongoing clinical trials are investigating combinations of CDK4/6 inhibitors with PI3K or AKT inhibitors in patients with advanced ER^+^/HER2^−^ breast cancer, reflecting the strong mechanistic potential for dual pathway targeting (Figure 3), although toxicity and tolerability remain important clinical considerations [115,116,117].

Compensatory activation of alternative cyclin-dependent kinases such as CDK2 and CDK7 is another mechanism of resistance [118]. While CDK4/6 inhibitors maintain RB in a hypophosphorylated, active state to force G1 arrest, upregulation of the cyclin E-CDK2 axis can restore RB phosphorylation and permit S-phase entry independent of CDK4/6 activity [8,119]. In parallel, CDK7, a component of the CDK-activating kinase complex and regulator of transcriptional control, can enhance cell cycle progression through modulation of both CDK activation and RNA polymerase II-dependent transcription [120,121]. Preclinical studies suggest that targeting these alternative CDKs may help overcome CDK4/6 inhibitor resistance by reinforcing durable proliferative arrest and preventing cell cycle re-entry in tumour cells (Figure 3) [121,122].

In addition to PI3K/AKT and alternative CDK activation, aberrant mitogenic signalling through the RAS-RAF-MEK-ERK (MAPK) pathway has also been implicated in resistance to CDK4/6 inhibition (Figure 3). The activation of the MAPK pathway promotes proliferation and survival independent of CDK4/6 activity [104,123]. Upregulation of MAPK signalling can sustain transcriptional programs that support cell cycle progression despite sustained RB hypophosphorylation [124]. Several ongoing phase I and phase II clinical trials are investigating combined CDK4/6 inhibition and MEK inhibition in breast cancer, as well as in other malignancies such as colorectal cancer, melanoma, and non-small cell lung cancer [125]. One finished trial is the PALBOBIN trial, which demonstrated substantial toxicity and limited efficacy in advanced pre-treated, triple-negative breast cancer, despite earlier preclinical data suggesting potential benefits, and that this combination should not be further developed in this disease [126]. In contrast, studies such as the MULAN trial in luminal breast cancer with multiple combinatorial approaches, including MEK inhibition, may further clarify the clinical therapeutic potential of MAPK inhibition in hormone receptor-positive disease [125,127].

Building on these immune-modulatory effects such as enhanced antigen presentation, Treg suppression, and paradoxical PD-L1 upregulation, the combination of CDK4/6 inhibitors with immune checkpoint inhibitors represents a mechanistically grounded strategy [76,128,129]. This observation provides a mechanistic basis for combining CDK4/6 inhibitors with anti-PD-L1 agents to enhance anti-tumour immune responses (Figure 3) [76,130]. Collectively, these findings illustrate that CDK4/6 inhibition extends beyond cell cycle control, influencing tumour signalling networks and immune dynamics within the tumour microenvironment.

As previously discussed, CDK4/6 inhibitors induce senescence, and although initially tumour-suppressive, they may contribute to resistance development in cancer cells over time [131]. Therefore, targeting senescent cancer cells represents a therapeutic opportunity to potentially reduce the risk of resistance. This concept is encapsulated in the “one-two punch” approach (Figure 3), whereby senescence is first induced in cancer cells using CDK4/6 inhibitors, followed by the administration of senolytic agents to selectively eliminate senescent cancer cells [132,133]. CDK4/6 inhibitor-pretreated breast cancer cells have been shown to exhibit increased vulnerability to lysomotropic agents owing to enhanced lysosomal activity [53], which is a hallmark of senescence. Consequently, lysosomal-targeting drugs can selectively eliminate those senescent breast cancer cells, supporting the notion of senescence as a therapeutically targetable state. This strategy may enable the removal of drug-tolerant and SASP-producing persister cells, thereby reducing the risk of tumour recurrence and microenvironment-driven resistance [53]. Beyond breast cancer, senolytic strategies have shown promise in other tumour types, including HPV-negative head and neck squamous cell carcinoma and triple-negative breast cancer, where palbociclib-induced senescence sensitises cells to navitoclax through BCL-xL upregulation, with a galacto-conjugated navitoclax prodrug further improving selectivity by exploiting elevated SA-β-galactosidase activity in senescent cells [134,135]. In colorectal cancer, MCL1 was identified as the key senolytic target via CRISPR screening, and its inhibition alongside palbociclib additionally reduced PD-L1 expression and restored CD8+ T cell cytotoxicity [136]. However, this approach is context-dependent, as in pleural mesothelioma, palbociclib induces only reversible pseudo-senescence, rendering cells insensitive to senolytics and highlighting the need to verify the permanence of therapy-induced senescence before pursuing such combinations [137]. These findings suggest that lasting tumour control will likely require combination strategies that not only target compensatory proliferative signalling but also account for the senescent cell populations that persist under CDK4/6 inhibition and contribute to microenvironmental resistance.

## 6. Discussion and Future Perspectives

CDK4/6 inhibitors were originally developed to induce cell cycle arrest at the G1-S transition phase through RB protein hypophosphorylation and the suppression of cancer cell proliferation [138,139]. However, their effects extend beyond transient cytostatic arrest [140]. Sustained RB activation represses E2F-driven transcription and promotes chromatin remodelling at proliferation-associated promoters, resulting in a durable transcriptional silencing [141,142]. Importantly, with prolonged exposure, this state can transition into senescence characterised by stable cell cycle arrest, the activation of tumour suppressor pathways, and the development of a partial SASP [26]. Notably, this SASP appears more restrained than that induced by DNA-damaging therapies, with reduced pro-inflammatory signalling, while preserving immune-modulatory functions [26]. These findings position CDK4/6 inhibitors as inducers of a distinct cellular state rather than pure cytostatic agents. CDK4/6 inhibitor-induced senescent cells remain metabolically active and modulate the tumour microenvironment through partial SASP secretion [26,70]. Despite substantial clinical success, the emergence of resistance and the need for more precisely targeted combination strategies define the future development of CDK4/6 inhibitor therapies. Next-generation CDK4/6 inhibitors, such as atirmociclib, are being investigated for better selectivity with less toxicity [50]. Dalpiciclib has also yielded promising efficacy, as its combination with fulvestrant in the phase 3 DAWNA-1 trial significantly prolonged progression-free survival in patients with advanced HR^+^/HER2^−^ breast cancer [4]. These findings highlight the importance of optimised combination strategies to extend clinical benefits. Importantly, CDK4/6 inhibitor-induced senescence in cancer creates exploitable therapeutic vulnerabilities. Senescent breast cancer cells exhibit increased lysosomal activity and metabolic reprogramming, rendering them more sensitive to lysosomotropic agents [53]. Together with chromatin remodelling and partial SASP production, these features define a targetable cellular state that may be leveraged through sequential treatment approaches. Overall, CDK4/6 inhibitor-induced senescence represents a central determinant of therapeutic response. A deeper understanding of its regulation, persistence, and context-specific effects will be essential for optimising CDK4/6-based therapies and developing more precise mechanism-driven treatment strategies.

## 7. Conclusions

CDK4/6 inhibitors extend beyond cytostatic arrest to induce a distinct senescent state in both tumour and normal cells, characterised by a context-dependent SASP that shapes immune responses, stromal interactions, and cancer cell plasticity. The restricted p53-dependent secretory profile associated with CDK4/6 inhibitor-induced senescence distinguishes it from classical DNA damage-induced senescence and may limit adverse effects in normal tissues. Senescence-directed combination strategies, including sequential senolytic approaches, offer promising avenues to eliminate residual senescent tumour cells and reduce recurrence risk. A deeper understanding of the regulation and persistence of CDK4/6 inhibitor-induced senescence will be essential for developing more precise treatment strategies.

## Data Availability

No new data were generated or analysed in this study.
